# A Case Report of a Child With Rare Phosphatidylinositol Glycan Anchor Biosynthesis Class N (PIGN) Gene Mutation With Hypotonia, Epilepsy, and Global Developmental Delay

**DOI:** 10.7759/cureus.80072

**Published:** 2025-03-05

**Authors:** Haydy M Khalifa, Haya Alkayyat, Raafat Hamad Seroor H Jadah

**Affiliations:** 1 General Medicine, Bahrain Defense Force Military Hospital, Riffa, BHR; 2 Pediatrics, Bahrain Defence Force Royal Medical Services, Riffa, BHR; 3 Pediatric Neurology, Bahrain Defense Force Military Hospital, Riffa, BHR

**Keywords:** autosomal recessive genetic disorder, epilepsy in children, global developmental delay (gdd), hypotonia, pign gene

## Abstract

Phosphatidylinositol glycan anchor biosynthesis class N (PIGN) gene mutation is a rare autosomal recessive genetic disorder. PIGN is essential for the glycosylphosphatidylinositol (GPI) anchor biosynthesis pathway. These mutations are linked to multiple congenital anomalies-hypotonia-seizures syndrome 1 (MCAHS1). The affected PIGN gene leads to a significant reduction in the overall levels of GPI-anchored proteins and CD24 expression, suggesting that even partial depletion of these proteins can result in severe phenotypic manifestations.

We present a case of a two-year-old boy diagnosed with spastic cerebral palsy following a hypoxic insult, who also exhibited refractory epilepsy, global developmental delay, and failure to thrive. Whole-exome sequencing confirmed a diagnosis of an autosomal recessive mutation in the PIGN gene.

Given that mutations in the PIGN gene may be an underrecognized cause of epilepsy, this case report aims to highlight the importance of early diagnosis of this condition. Furthermore, our findings contribute to the expanding spectrum of PIGN gene mutations.

## Introduction

Mutations in the phosphatidylinositol glycan anchor biosynthesis class N (PIGN) gene are the hallmark of this uncommon autosomal recessive genetic condition. The PIGN gene encodes a protein essential for the formation of glycosylphosphatidylinositol (GPI) anchors, which are critical for anchoring proteins to the cell surface in many blood cells. This protein, expressed in the endoplasmic reticulum, facilitates the transfer of phosphoethanolamine (EtNP) to the first mannose of the GPI anchor [1,2].

More than 20 genes are involved in the GPI-anchor biosynthesis pathway, with PIGN specifically responsible for adding EtNP to the core mannose structure. Mutations in the PIGN gene have been linked to multiple congenital anomalies-hypotonia-seizures syndrome 1 (MCAHS1) [3].

MCAHS1 is an autosomal recessive condition characterized by epilepsy, growth restriction, hypotonia, and other congenital anomalies. It is associated with mutations in the PIGN gene. Additionally, numerous neurological disorders have been linked to mutations in the PIGN gene, which plays a crucial role in the GPI-anchor pathway [4].

## Case presentation

The patient was a two-year-old child who was previously healthy until the age of two months when he presented with repeated episodes of generalized tonic-clonic convulsions lasting for a few minutes, associated with up-rolling eyes, frothy oral secretions, and circumoral cyanosis, followed by postictal drowsiness. The patient also showed regression in his developmental milestones, as he lost his social smile and eye fixation and began to exhibit poor head control and an inability to move.

He was born to consanguineous parents via full-term normal vaginal delivery at 40 weeks of gestation with an Apgar score of 9 and 9 at 1 and 5 minutes, respectively. His initial birth growth parameters were within the 50th percentile (birth weight: 3.84 kg, length: 53 cm, and head circumference: 36 cm). There was no family history of epilepsy or global developmental delay (GDD). On physical examination, he was constantly unconscious and non-responsive to surrounding sounds, when called by name, or when touched. He could maintain normal breathing in room air; no dysmorphic features or neurocutaneous skin lesions were observed. He has a nasogastric tube attached due to difficulty swallowing, and a percutaneous endoscopic gastrostomy tube is attached for feeding. He was unable to fix and follow and had continuous horizontal nystagmus. His muscle power was 2/5 in all limbs, with reduced muscle tone and brisk deep tendon reflexes (+4) associated with ankle clonus. The red reflex test was conducted, which appeared to be normal bilaterally. The rest of the systemic examination was unremarkable.

**Figure 1:**
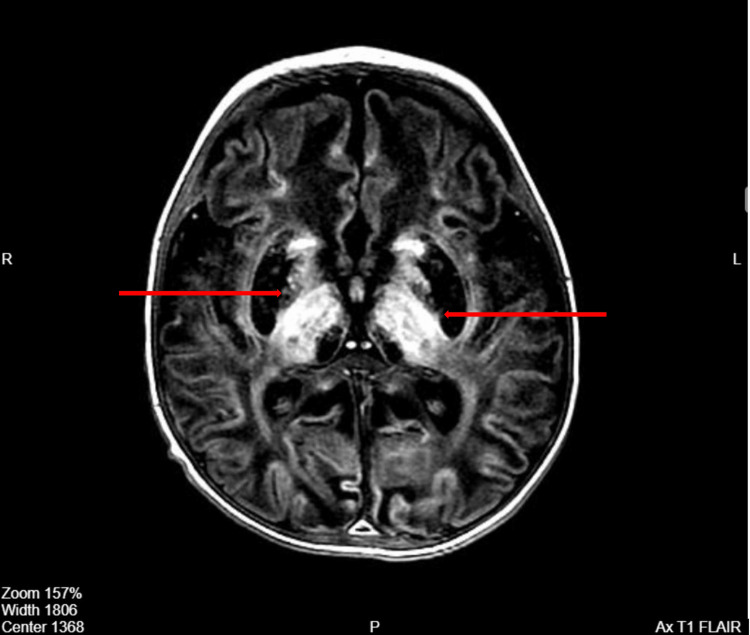
Brain MRI T1 FLAIR sequence showing abnormal signal intensity on the basal ganglia and thalami on both sides FLAIR: Fluid-attenuated inversion recovery

**Figure 2:**
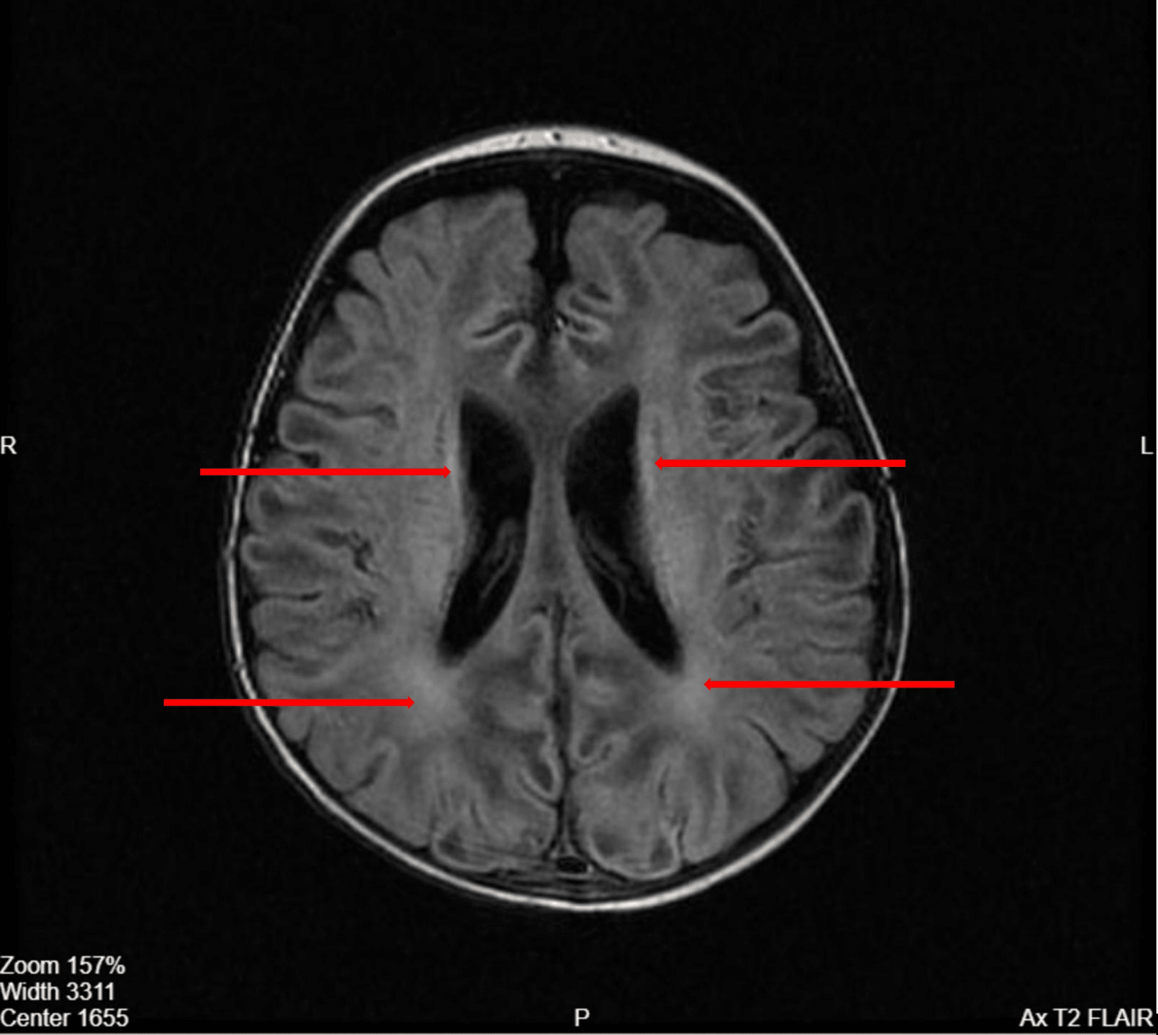
Brain MRI T2 FLAIR sequence showing abnormal signal intensity at the periventricular and occipital white matter on both sides FLAIR: Fluid-attenuated inversion recovery

**Figure 3:**
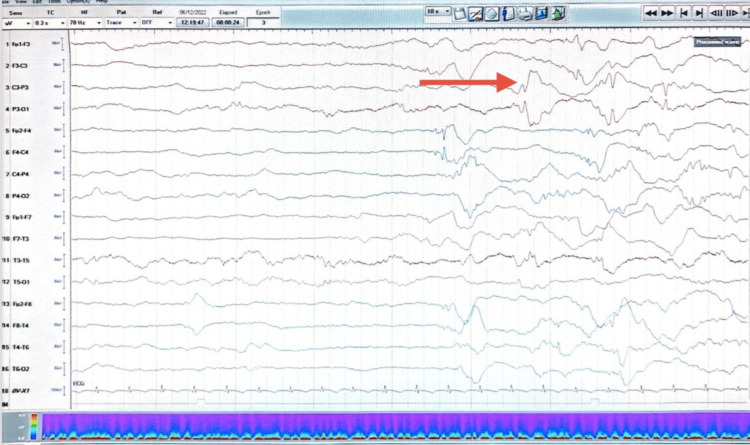
EEG showing left parietal epileptiform sharp transient discharge

**Figure 4:**
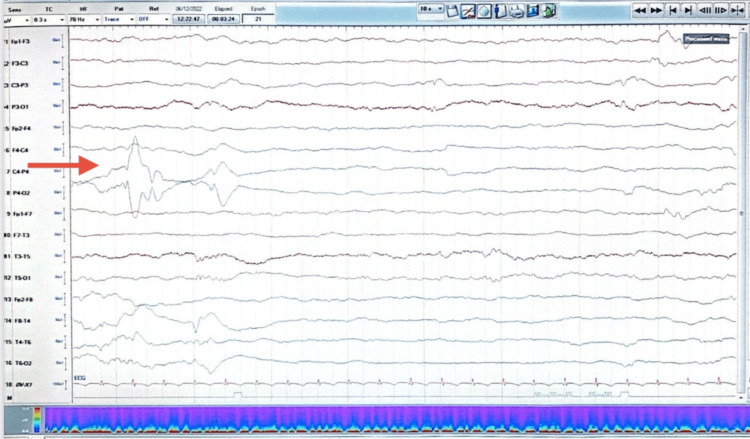
EEG showing right parietal epileptiform sharp transient discharge

His initial brain magnetic resonance imaging (MRI) showed hyperintense signal intensities within the basal ganglia, thalami, and bilateral cortical gyri on T2-weighted and fluid-attenuated inversion recovery (FLAIR) MRI sequences (Figures 1, 2). His electroencephalogram (EEG) was abnormal, revealing bi-parietal epileptiform sharp transient discharges (Figure 3, 4). The patient also underwent whole-exome sequencing (WES), which confirmed a diagnosis of a PIGN mutation.

He was started on antiepileptic medication, levetiracetam (Keppra), with no further seizure attacks. However, he exhibited further regression in his developmental milestones, with a complete loss of social interaction and significant motor delay, becoming unable to sit or walk. The patient is under regular follow-up with a multidisciplinary team, including a general pediatrician, pediatric neurologist, orthopaedic surgeon, physiotherapist, and dietitian. 

## Discussion

PIGN gene mutation is a rare autosomal recessive syndrome characterized by dysmorphic features and multiple congenital anomalies, along with severe neurological impairment, chorea, and seizures, ultimately leading to early death. The identification of a gene involved in the pathogenesis of this disease has been crucial. The varied phenotypic characteristics of affected patients and the involvement of multiple body systems are consistent with the high expression of PIGN in different organs [5].

Mutations in PIGN can lead to visceral abnormalities (>90%), psychomotor developmental delay (100%), hypotonia (100%), and seizures (93%). Such mutations may be lethal in the fetal or neonatal stage and can contribute to various congenital abnormalities, including hypotonia-seizures syndrome, and Fryns syndrome [6] is a condition that affects multiple body systems, most commonly causing a congenital diaphragmatic hernia.

Signs and symptoms of PIGN mutations typically manifest during pregnancy or within the first few months of life. The broad range of clinical characteristics is due to the widespread expression of PIGN in many tissues. Neonatal hypotonia, seizures, delayed or absent psychomotor development, and other congenital abnormalities are the hallmark features of PIGN [7,8].

Neurological symptoms include intellectual impairment, brain atrophy, epileptic seizures, hypotonia, hyporeflexia, and hyperreflexia. Congenital defects may affect the urinary tract, diaphragm, heart (atrial septal defect, persistent foramen ovale), and gastrointestinal system (gastroesophageal reflux). Facial abnormalities associated with PIGN mutations include cleft palate, narrow lips, a small nose, a depressed nasal bridge, epicanthal folds, large fleshy ears, low-set ears, a long philtrum, and micrognathia (small lower jaw) [7,8].

The diagnosis of PIGN mutations is primarily based on the patient’s clinical presentation, including GDD, hypotonia, and epilepsy. However, WES is considered the gold standard for diagnosing rare genetic disorders [9].

The functional severity of PIGN mutations appears to correlate with the clinical severity of the condition. The extent of congenital abnormalities may also depend on the severity of the mutations. Additional genetic and environmental factors should be considered. It is important to note that some tissues are more sensitive than others to PIGN-related impairment during development. In the case of this patient, no additional congenital abnormalities were observed in the gastrointestinal, cardiac, or urinary systems. The patient also did not exhibit gastroesophageal reflux, diaphragmatic hernia, brachycephaly, a flat face, hypoplasia of the distal digits, an open mouth, or excessive drooling. Patients with epilepsy from diverse ethnic backgrounds should be evaluated for MCAHS1 or PIGN mutations, as these may be responsible for PIGN-related epilepsy [10].

The PIGN mutation can only be effectively treated through genetic therapy. However, as no such therapy is currently available, treatment remains limited to supportive and symptomatic management. Due to the associated complications and the patient's ongoing developmental delays, the prognosis remains poor.

## Conclusions

This is a rare autosomal recessive genetic condition caused by a PIGN gene mutation, presenting with GDD, hypotonia, and epilepsy. WES is essential for patients with unexplained GDD associated with epilepsy. Alternatively, a stepwise genetic approach can be utilized to detect such genetic mutations, including targeted gene panels or exome sequencing, as a more cost-effective option. The clinical severity of the illness is most likely related to the extent of protein truncation. Each individual's expressed phenotype helps us understand this hereditary disease. This case report aims to contribute to the existing knowledge about PIGN gene mutation.
